# FRAMA: from RNA-seq data to annotated mRNA assemblies

**DOI:** 10.1186/s12864-015-2349-8

**Published:** 2016-01-14

**Authors:** Martin Bens, Arne Sahm, Marco Groth, Niels Jahn, Michaela Morhart, Susanne Holtze, Thomas B. Hildebrandt, Matthias Platzer, Karol Szafranski

**Affiliations:** Leibniz Institute on Ageing - Fritz Lipmann Institute, Beutenbergstr. 11, 07745 Jena, Germany; Leibniz Institute for Zoo and Wildlife Research, Alfred-Kowalke-Straße 17, 10315 Berlin, Germany

**Keywords:** RNA-seq, Transcriptome assembly, Full-length mRNA, Naked mole-rat

## Abstract

**Background:**

Advances in second-generation sequencing of RNA made a near-complete characterization of transcriptomes affordable. However, the reconstruction of full-length mRNAs via de novo RNA-seq assembly is still difficult due to the complexity of eukaryote transcriptomes with highly similar paralogs and multiple alternative splice variants. Here, we present FRAMA, a genome-independent annotation tool for de novo mRNA assemblies that addresses several post-assembly tasks, such as reduction of contig redundancy, ortholog assignment, correction of misassembled transcripts, scaffolding of fragmented transcripts and coding sequence identification.

**Results:**

We applied FRAMA to assemble and annotate the transcriptome of the naked mole-rat and assess the quality of the obtained compilation of transcripts with the aid of publicy available naked mole-rat gene annotations.

Based on a de novo transcriptome assembly (Trinity), FRAMA annotated 21,984 naked mole-rat mRNAs (12,100 full-length CDSs), corresponding to 16,887 genes. The scaffolding of 3488 genes increased the median sequence information 1.27-fold. In total, FRAMA detected and corrected 4774 misassembled genes, which were predominantly caused by fusion of genes. A comparison with three different sources of naked mole-rat transcripts reveals that FRAMA’s gene models are better supported by RNA-seq data than any other transcript set. Further, our results demonstrate the competitiveness of FRAMA to state of the art genome-based transcript reconstruction approaches.

**Conclusion:**

FRAMA realizes the *de novo* construction of a low-redundant transcript catalog for eukaryotes, including the extension and refinement of transcripts. Thereby, results delivered by FRAMA provide the basis for comprehensive downstream analyses like gene expression studies or comparative transcriptomics. FRAMA is available at https://github.com/gengit/FRAMA.

**Electronic supplementary material:**

The online version of this article (doi:10.1186/s12864-015-2349-8) contains supplementary material, which is available to authorized users.

## Background

Since decades, characterization of transcriptomes by random sequencing of cDNA has been practiced to decipher the gene repertoire for a large number of organisms [1–4]. The resulting compilation of mRNA sequences, a so-called transcript catalog, is an important fraction of the functional genetic information and serves as a basis for multiple downstream analyses including gene expression studies, using either microarray techniques or tag sequencing, as well as comparative sequence analyses [5, 6]. Particularly, the full-length protein-coding sequence (CDS) represents a crucial entity forming a knowledge base in genetics research [7]. Fragmentary information will lead to incomplete, ambiguous, or even mislead conclusions in downstream analyses. While in principle, a genome-wide catalog of CDSs can also be derived from a genome sequence using gene prediction programs, it is nowadays a standard to support gene predictions with mRNA sequence evidence [8–11]. Transcriptome sequencing is also able to characterize untranslated regions (UTRs) [12], which cannot be predicted from the genome ab initio. UTRs include the landing platforms for potential regulatory interactions with micro-RNAs and, in combination with genomic sequence, also allow definition of promoter regions, both of which are important for functional gene analysis.

While the introduction of second-generation sequencing of RNA (RNA-seq) made the characterization of transcriptomes very affordable, the short-read RNA-seq data cannot display mRNA molecules in their entirety. Therefore, assembly programs were designed to reconstruct, as good as possible, full-length mRNA sequences from short RNA-seq reads [13, 14]. While these assembly programs have reached an accepted level of quality, they still face severe difficulties. The sequence depth of RNA-seq may be sufficient to detect rare mRNAs but, often, is still too low to allow reconstruction of their entire structure, which results in fragmented transcript contigs. In addition, eukaryotic transcriptomes are very complex by showing several alternative splice variants per gene, multiple gene copies, single nucleotide polymorphisms and transcribed pseudogenes. It is noteworthy that, for protein-coding genes, even the most highly expressed transcript is not necessarily protein-coding [15].

Functionally relevant signatures of non-model organisms in comparison to related organisms, such as gene content and transcript structures, can be read out most conveniently using a low redundancy subset of the transcript assembly. Identification of this representative assembly subset is possible by orthologous inference. In the past, complex algorithms have been developed for genome-wide identification of orthologous and homologous groups between different species [16]. Nevertheless, best available contigs may still show peculiarities, such as incompleteness, retained introns or splicing variants with premature stop codons. Additionally, overlapping genes may result in fusion contigs [17]. Thus, starting from de novo transcriptome assembly, strategies are required to scaffold fragmented contigs, to isolate single transcripts from fusion contigs, and to select or correct contigs in order to show the likely protein-coding transcript variant. Several of these illustrated tasks have been previously addressed in the course of project-specific assembly/annotation projects [18–21], but were not yet incorporated into re-useable software concepts.

Here, we present a genome-independent software tool (FRAMA) that specifically addresses post transcript assembly tasks for eukaryote transcriptomes. These tasks include reduction of assembly redundancy, ortholog-based gene symbol assignment, correction of fusion transcript contigs and scaffolding of fragmented transcript contigs, CDS identification and clipping of weakly supported sequence termini. We applied this pipeline to de novo assembly and annotation of the transcriptome of the naked mole-rat (NMR; *Heterocephalus glaber*), the longest-living rodent known and a promising non-model organism in ageing research [22, 23]. Two independent NMR genome assemblies and associated gene annotations are available [24, 25] and were used for a validation of our pipeline results. The comparison of the different approaches for gene model construction indicates that FRAMA is competitive and fulfills accepted quality standards.

## Implementation

**Fig. 1:**
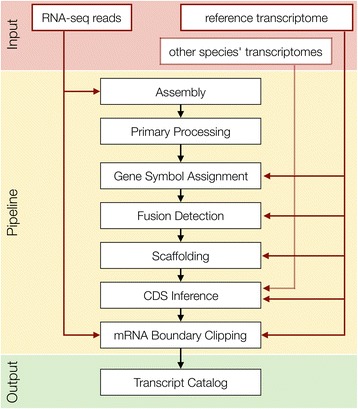
Stages of the FRAMA procedure. Black arrows show the flow of data, red arrows indicate which stages make use of input data, and light red arrows indicate optional use of input data

FRAMA is a novel software suite that calls components written in Perl and external software (Additional file 1: Table S1), applicable on UNIX/Linux and MacOS computer systems. Mandatory required input are RNA-seq read data, either paired-end or single-end, strand-specific or non strand-specific, and a comprehensively annotated transcriptome of a related species. FRAMA executes in 8 successive steps: (i) assembly, (ii) primary processing, (iii) gene symbol assignment, (iv) fusion detection, (v) scaffolding, (vi) identification of CDS, (vii) identification of mRNA boundaries, and (viii) descriptive assembly statistics (Fig. 1). Software parameters for each step can easily be edited in a parameter file. FRAMA produces a representative compilation of transcripts, a so-called transcript catalog, with CDSs and mRNA boundaries annotated. In the transcript catalog, each transcript will have a one-to-one relationship to an orthologous transcript in the reference transcriptome.

### Assembly and primary processing

A variety of de novo transcriptome assembly tools are available, which perform differently well on separate subsets of transcripts [14]. FRAMA currently utilizes Trinity, an allrounder that performs well across different species and library properties [13, 18, 19]. Trinity starts with a greedy assembly of linear contigs based on the most frequent k-mers to reconstruct one full-length isoform per locus and additional unique regions partially. Then, overlapping contigs are clustered and connected into a de Bruijn graph, which represents different alternative splice variants for one locus or highly similar homologs. Finally, Trinity reports contig sequences that represent probable paths through each graph [13].

NCBI recommends scanning of transcript assembly data for adapter, vector and other cross-project contaminations that might occur. Accordingly, FRAMA examines the final scaffolded and annotated transcriptome for vector contamination using NCBIs VecScreen criteria [26], and match regions are annotated with match score and topological category.

Redundancy among transcript contigs can arise from shorter transcript contigs which are fully embedded in longer contigs or from local differences arising from sequencing errors or allelic variations. In order to reduce redundancy, in an optional step, transcript contigs are clustered using CD-HIT-EST. The cluster will then be replaced by the longest representative contig. Additionally or alternatively, TGICL can be used to combine overlapping transcript contigs into single longer contigs. Order of execution of both software programs can be chosen arbitrarily.

### Assignment of gene symbols

Gene symbol assignment to transcript contigs is performed on the nucleotide level, based on best bidirectional BLASTN hits (BBH) against CDSs of an orthologous reference transcriptome. This enables the most sensitive differentiation of paralogous proteins. For example, the genes *CALM1*, *CALM2* and *CALM3* express identical proteins, in the NMR and other mammals, but differ in their CDS (Additional file 2: Figure S1). As an additional advantage of the nucleotide-level search, the identification of CDS for BLASTP or more time-consuming BLASTX searches are not necessary. Following the gene symbol assignment based on BBHs, remaining unassigned transcript contigs that show a single best hit (SBH) to an unassigned reference transcript are labeled and added to the transcript catalog. Annotated transcript contigs become oriented according to its assigned ortholog, which is essential if unoriented read data are used for assembly.

Finally, all annotated transcript contigs are examined for further BLAST hits, which may overlap with the initially identified orthologous region. This identifies “misassembled” contigs, which presumably originate from chimeric cDNA as well as neighboring or overlapping genes. The contigs that contain multiple genes are copied to represent each gene separately, which allows independent processing of the genes in subsequent processing steps.

### Scaffolding

**Fig. 2:**
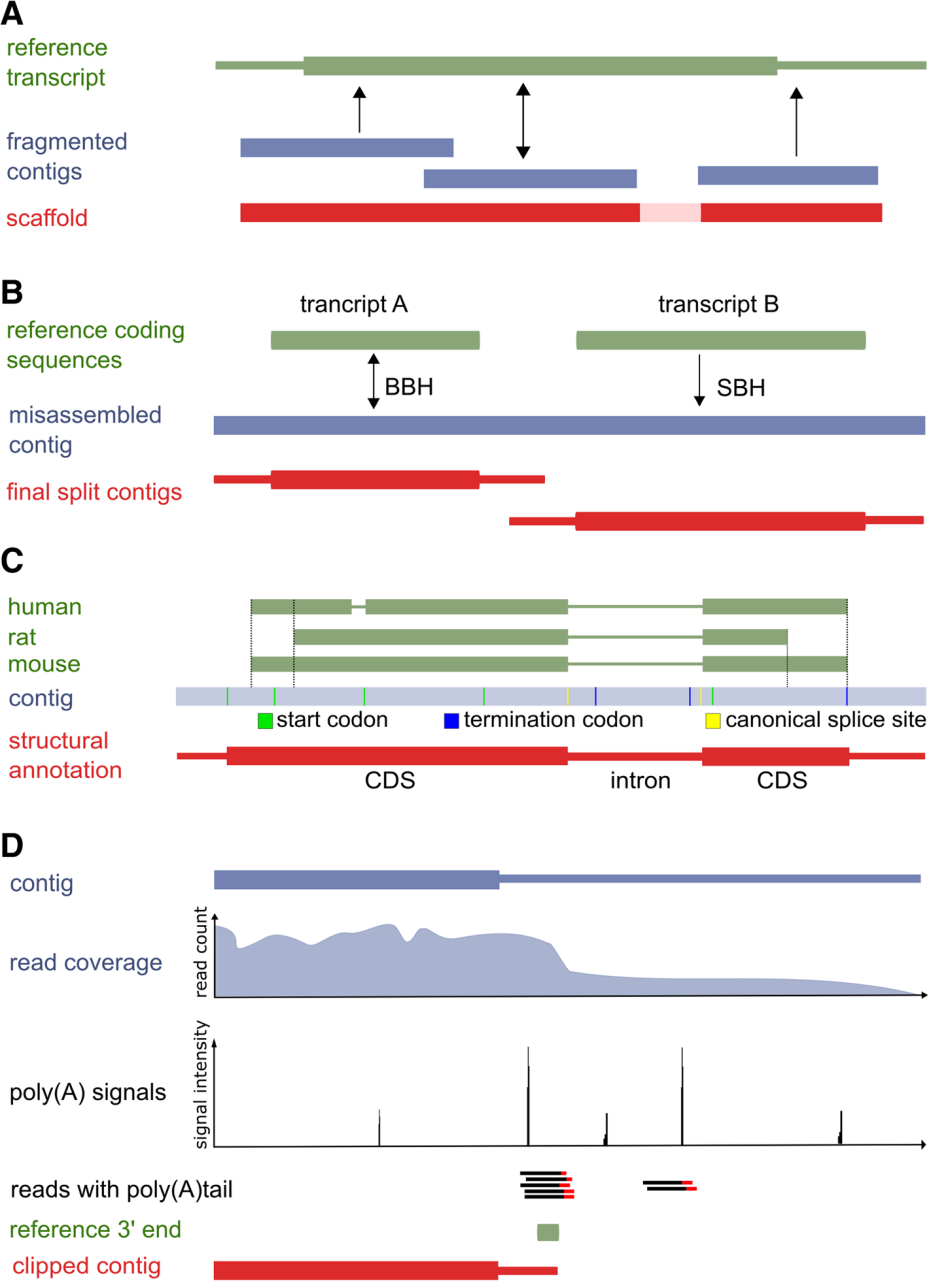
Schematic illustration of complex processing stages in FRAMA: **a** inference of CDS using orthologous transcripts from related species; **b** ortholog-based detection of fusion contigs; **c** scaffolding; **d** clipping of transcript 3’ termini by the use of weighted scores for indicative features. Horizontal bars indicate contigs and mRNAs, thicker regions indicate CDS. Colors code the origin of sequence data: Trinity contig (*blue*), orthologous transcript (*green*), final FRAMA transcript (*red*)

FRAMA performs an ortholog-based scaffolding of fragmented transcript contigs (Fig. 2). To achieve this, FRAMA uses transcript contigs without an assigned gene symbol, but with BLASTN hits to previously identified orthologous counterparts. These candidate transcript contigs are then aligned to the orthologous counterpart using MAFFT. Next, the minimum number of fragments spanning most of the reference transcript is determined using a greedy algorithm. Finally, the core contig sequence is extended by the series of winning candidates. Any gap between non-overlapping contigs is filled with an N stretch, whose size corresponds to the size of the orthologous transcript region.

### Identification of CDS

In order to identify the CDS, each FRAMA transcript is aligned with orthologous CDSs from the reference transcriptome and, optionally, other species as provided by an ortholog table (Fig. 1). Coordinates of each CDS are transferred to the transcript contig and examined for a valid CDS among all reading frames (Fig. 2). In the first course, a candidate reading frame should fit this orthologous window without premature stop codon or, in case of selenoproteins, without non-UGA stop codons. In the presence of multiple valid coding regions, the most complete one in respect to its corresponding ortholog is chosen. If the described approach fails, the CDS prediction (GENSCAN) that is most similar to that of the assigned ortholog is annotated. As a last resort, the longest ORF computed by EMBOSS GETORF is assigned.

### Identification of mRNA boundaries

As mentioned above, neighboring or overlapping genes could result in a single long contig and consequently need to be shortened to obtain one transcript contig corresponding to the assigned gene only. Furthermore, Trinity has difficulties determining the precise end of 3’ ends, in particular due to the imprecise cellular mechanism of 3’ end cleavage, alternative poly(A) sites or possible genomic contamination. Fortunately, mRNA 3’ termini share significant sequence conservation between species, e.g., human and mouse [27], and further evidence like poly(A) signal motifs and poly(A)-containing reads are used to infer more precise 3’ ends. Specifically, FRAMA scores potential 3’ ends according to the occurrence of poly(A) signals. Additionally, informative drops in read coverage as well as reads that contain protruding poly(A) sequence are identified via re-alignment of the RNA-seq data. Finally, a local alignment with 50 bp of the orthologous mRNA terminus is computed with EMBOSS needle. Each contig position is assigned a weighted score based on all four features using fuzzy logics, and clipping is applied at the most reliable position, using an empirically validated threshold. If GENSCAN predicts a promoter sequence, 5’ ends are clipped as well. In case of extra CDS regions that are predicted by GENSCAN and supported by a BLAST hit, clipping is always applied, either according to the scoring scheme or, if no reliable position was identified, at the center of intercoding regions.

## Results

### Sequencing

A limited overview of a tissue’s mRNA content could be obtained from assembly of 20 million RNA-seq reads preferably 100 nt or longer [28]. For a near-complete picture of a multi-cellular eukaryote, well over 100 million RNA-seq reads and a diversified tissue sampling are desirable, in order to recover tissue-specific genes and genes which are generally low in expression. For an application of FRAMA, we chose the latter concept and obtained strand-specific Illumina RNA-seq data from ten different tissues of the NMR (Additional file 1: Table S3). After quality filtering and joining of overlapping paired-end reads, the data consisted of 352.5 million single-end fragments with an average length of 194 bp (67.9 Gb in total). For quality control, reads were aligned to the NMR genome sequence, resulting in 90.9–96.2 % mapped reads per sample. Mapping rates above 90 % are comparably high and indicate good base quality of the RNA-seq data and good correspondence between RNA-seq data and the genome sequence [29]. Taking a curated set of NMR transcripts (TCUR), we could further validate that the dUTP protocol for RNA-seq is highly strand-specific. At least 99.85 % of mapped reads had the correct orientation.

### Assembly and primary processing

Read data from the ten tissue samples were used as pooled input to Trinity/FRAMA. The use of pooled samples was shown to improve the completeness of transcript contigs in contrast to merging of sample-specific assemblies [18]. The resulting raw assembly comprised 660,649 individual graphs, which, theoretically, reflect the number of assembled gene loci, and 1,042,649 transcript contigs. The length of contigs ranged from 200 bp, the default threshold of Trinity, up to 32,980 bp, with an N50 of 2687 bp (Additional file 1: Table S5).

Trials on meta-assembly indicate that both, CD-HIT-EST and TGICL do minor reductions (8.6 and 11.4 %, respectively) of the transcript contig set while an impact on the final transcript catalog is undetectable. Intending most conservative processing of the NMR data, we chose to continue with the primary Trinity assembly and in order to avoid false assemblies, e.g., collapsing of paralogs or joining of neighboring genes.

One step of sequence post-processing is the clipping of putative sequencing adapters from contig ends, which may show up even if adapter clipping was performed on the input RNA-seq data (0.04 % of contigs). Moreover, FRAMA scans transcript contigs for putative vector contamination, as recommended by the NCBI. As might be expected for the in vitro-cloned RNA-seq libraries, the sequence data is free of cloning vectors. However, NCBI VecScreen indicated 8 strong and 26 moderate vector hits, which we all classified as false positives upon thorough inspection. For example, vector pSOS (acc. no. AF102576.1) contains a fragment of human *SOS1* which produces a strong hit to the *SOS1* transcript of the NMR. Unfortunately, masking of these regions is required for submission to the NCBI Transcript Shotgun Assembly archive.

### Assignment of gene symbols

We chose human as the reference organism since the human gene annotation has superior quality and, in terms of sequence similarity, it is closer to the naked mole-rat than mouse, which has a gene annotation of similar quality (Additional file 1: Table S4). Using 34,655 human protein-coding reference transcripts (19,178 genes), FRAMA was able to identify 21,984 NMR counterparts, corresponding to 16,887 genes in total (88.0 % of human genes). The longest NMR transcript contig (32,980 bp) corresponds to the longest human gene, titin.

In general, transcripts that could not be identified in the NMR have much lower expression levels in human tissues, compared to those which could be identified (Additional file 2: Figure S2). For example, reconstructed versus non-reconstructed genes show 1301-fold higher median expression in human liver, and 396-fold higher expression in human kidney (both p < <0.001, Mann-Whitney *U* test). On the other hand, some highly expressed genes in human liver lack orthologs in the NMR. However, several of these were identified as primate-specific genes. For example, the top-expressed orphan human genes comprise three metallothionein genes (*MT1F*, *MT1H*, *MT1M*) which are part of the primate-specific expansion of the metallothionein-1 and -2 family [30]; four cytochrome P450 genes (*CYP2C8*, *CYP2C9*, *CYP2C19* and *CYP4F11*) which are primate-specific paralogs at multiple branches of the large family tree [31]; and factors of the major histocompatibility complex, *HLA-B* and *HLA-E*, which underwent fast evolution in primate populations [32].

### Scaffolding

Scaffolding was applied to 3684 FRAMA transcripts (3488 genes) and added 3.29 Mb sequence, resulting in a median information increase of 1.27-fold. We manually inspected 31 scaffolded FRAMA transcripts comprising 81 fragments in comparison to a curated set of NMR transcripts (TCUR) and determined errors in 5 scaffold fragments (6.2 %). Further, of all scaffolded FRAMA transcripts we identified only 111 (3.0 %) that show non-overlapping hits to multiple genome contigs in both genome assemblies. These failure rates likely represent the upper bound of errors since some of the non-validated scaffolds may result from fragmented genome data.

Following a series of physical processing steps from the initial Trinity assembly to pre-final transcript sequences, we sought to assess the completeness of the transcript catalog produced by FRAMA. For this we used CEGMA (Additional file 1: Table S6), a tool that identifies 248 eukaryotic core protein-coding genes and diagnoses their completeness. Since 245 genes scored “CDS complete” (98.8 %), the transcript sequence set produced by FRAMA appeared almost complete, within the performance range of other, genome-based transcript catalogs (TGNOMON 247, equivalent to 99.6 %; TKIM 237, 95.6 %; see Methods for definition of reference transcript sets). Interestingly, the initial Trinity transcriptome assembly contained even slightly less CEGMA genes (243 complete scores) than that of FRAMA, indicating that the final FRAMA output essentially encompasses all relevant genes contained in the initial assembly, and that subsequent processing steps even improved the recovery of the core gene set.

### Identification of CDS

**Fig. 3:**
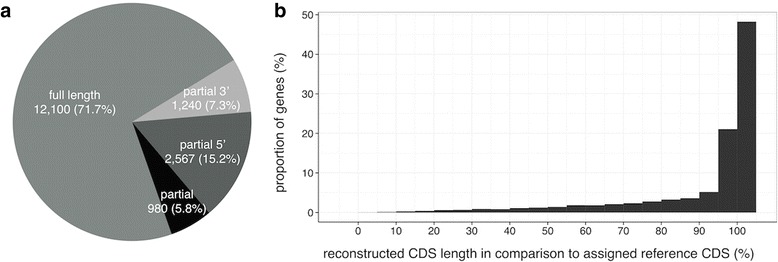
Completeness of CDS regions **a** classified according to ORF status, where “full length” refers to existing start and stop codons; **b** histogram of correspondence between (partly) recovered CDS and orthologous CDS

The majority of coding regions (13,841 genes; 82.0 %) were assigned with evidence from orthologous sequences. GENSCAN additionally identified CDS of 2765 genes, of which 26.4 % contained introns with canonical splice sites. Taken together, most resulting NMR genes had a full-length ORF including start and stop codon (12,100; 71.1 %; Fig. 3a). This is further supported by 12,583 genes (74.5 %) that had their CDS reconstructed over >90 % of the orthologous length (Fig. 3b). Correctness of the inferred CDS and the assigned gene symbol was validated by BLASTP searches against the human proteome, revealing 96.3 % of transcript contigs that hit proteins with the correct gene symbol, plus 2.9 % that gave hits to the same gene family.

### Identification of mRNA boundaries

**Fig. 4:**
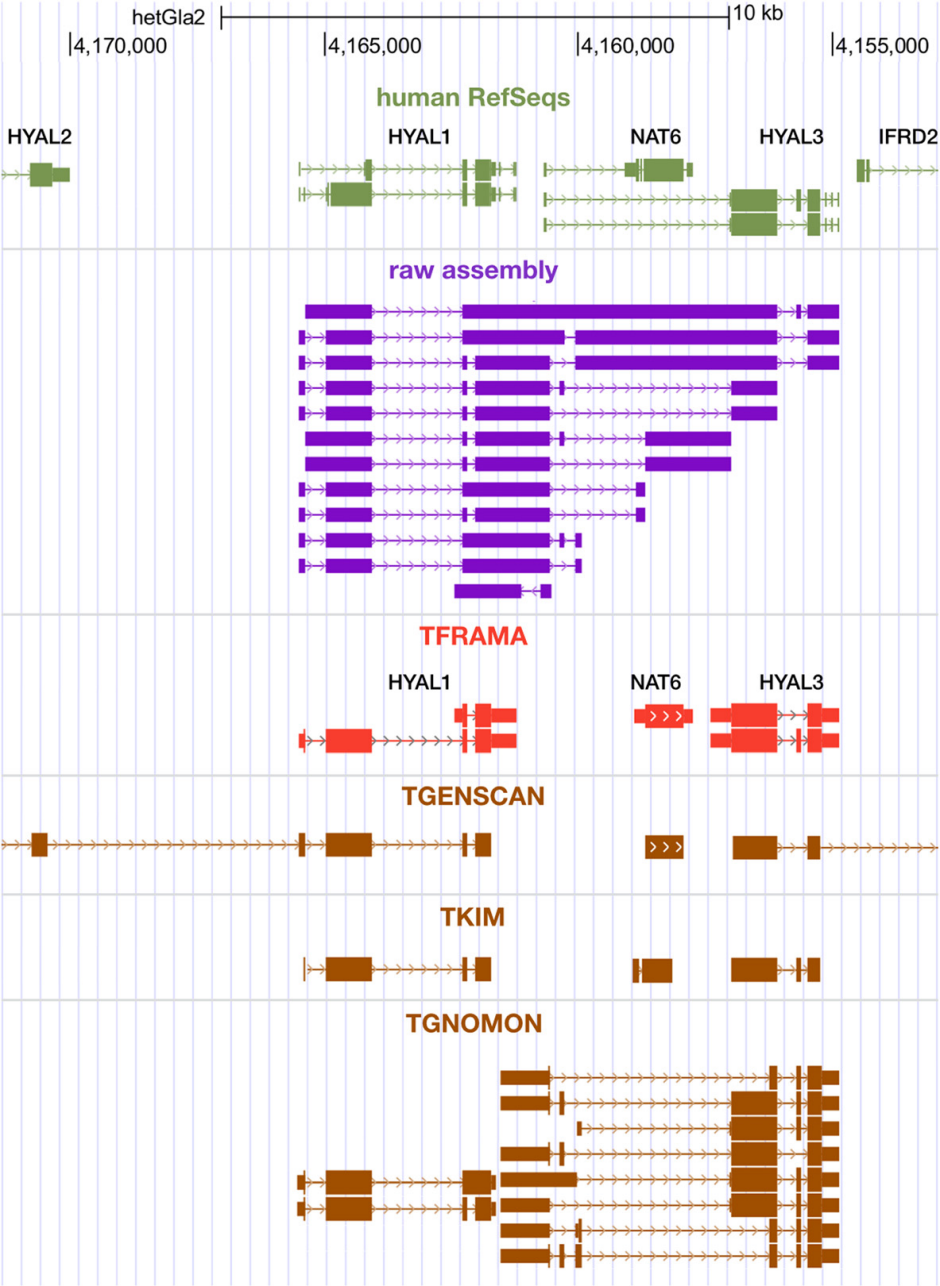
A genome-based transcript map showing misassembled Trinity contigs (*purple track*) and improvements made by FRAMA’s mRNA boundary clipping (*red track*). Human RefSeq counterparts to FRAMA transcripts are shown in green. Trinity provides a plethora of (putative) transcript isoforms (63 contigs) for the *HYAL1-NAT6-HYAL3* locus, many of them being read-through variants that join neighboring genes (informative subset in purple track). Although FRAMA is not able to resolve the shared first exon of the *NAT6*-*HYAL3* locus correctly, mRNA boundary clipping improved the raw assembly substantially by separating the gene loci. Genome-based methods (brown tracks) struggle in predicting the correct gene loci, too: TKIM shows the best performance, separating each gene locus correctly. GENSCAN correctly separates *HYAL1*, *NAT6* and *HYAL3* loci, but joins neighboring loci (*HYAL1* with *HYAL2* and *HYAL3* with *IFRD2*). GNOMON correctly provides several different *HYAL3* variants, but misses *NAT6* completely. Throughout the figure, thick bars represent coding regions, thin bars untranslated regions and lines introns. Arrows on lines or bars indicate the direction of transcription. Accession numbers of external gene models are listed in Additional file 1: Table S11

During gene symbol assignment, FRAMA identified 12 fusion transcript contigs that arose mostly from neighboring genes (Fig. 4). This does not reflect the total number of misassembled transcript contigs, because different misassembled variants have been assigned to different orthologous genes by the BBH/SBH strategy. In total, GENSCAN predicted multiple CDS for 1127 FRAMA NMR transcripts (5.1 %; 1069 genes). This is a higher proportion than seen on human and mouse RefSeq transcripts (3.5 and 2.6 %, respectively), which we consider as the background level of false positive GENSCAN predictions. Consistently, 52.4 % of the NMR transcripts with extra CDS predictions are supported by cross-species BLAST hits (591 transcripts, 516 genes) and thus likely result from correct CDS predictions. The remaining proportion of spurious predictions is comparable to the level in human and mouse transcripts. In total, summing the effect of all clipping procedures, FRAMA removed 5.13 Mb sequence from 5556 transcripts (4774 genes).

### Genome-based validation of transcript catalog

A recurring problem in the validation of de novo assemblies is the absence of a reference or gold standard. We chose to compare transcripts computed by FRAMA (TFRAMA) with publicly available NMR transcripts and gene annotations (Additional file 1: Table S7). We considered in-house curated transcripts (TCUR) that were reconstructed using a genome-independent approach as the gold standard in this comparison of NMR sequences. Two previous efforts provided NMR transcript catalogs based on a combination of ab-initio gene prediction, orthologous matching and RNA-seq evidence - one by Kim et al. reported transcript models (TKIM) [24] based on genome assembly hetgla1, and one computed RefSeq transcripts using NCBI’s GNOMON pipeline (TGNOMON) based on both available genome assemblies (hetgla1, hetgla2). Further, our validation included transcripts obtained only from ab initio prediction (TGENSCAN).

In transcript-genome alignments 96.8 % of TFRAMA could be aligned (92.7 % of sequence), but only 78.7 % of these transcripts were aligned over their entire length (>99 %). Since a realignment of TGENSCAN to its source genome gives 98.9 % of transcripts matching over their entire length (99.9 % of sequence), the technical error rate appears negligible. Interestingly, TCUR showed non-matching and mismatching regions with a rate depending on the genome sequence, 4.1 % exons on hetgla1, 1.0 % on hetgla2 (Additional file 1: Tables S8 and S9). However, 92.0 % of conflicting regions were validated by one genome version, which indicates that missing or discontinuous genome sequence are the source of conflicts with TCUR transcript models. We reject the possibility that genetic differences of the underlying NMR material explain the genome-transcriptome differences since well-aligned regions have very high sequence similarity, 99.9 % between TCUR and both genome versions and 99.9 % between TFRAMA and hetgla2. In conclusion, TFRAMA consistently fills missing and weak genome sequence. Effectively, TFRAMA-genome alignments spanned 1695 sequence gaps within scaffolds of hetgla2 and added 408,293 bp novel sequence.

We also validated the consistency of transcript sets, using the RNA-seq data produced in this study, by calculating the proportion of transcript-genome alignments covered by reads (coverage breadth). As expected, the majority of TFRAMA (98.1 %) is completely supported by RNA-seq reads (transcripts with >95 % coverage breadth). In contrast, only 18.7 % of TGENSCAN are completely supported by reads, while 22.4 % are sparsely covered (<5 % coverage breadth). Evidence-based methods show better agreement with our experimental data (TGNOMON 87.6 %, TKIM 71.5 % completely supported).

We compared the transcript-genome alignments of TGNOMON, TKIM, TGENSCAN and TFRAMA with those of our gold standard data set, TCUR (Table 1, Additional file 2: Figure S3). All methods achieved a similar recovery rate of TCUR gene loci (TGNOMON 135, 99.3 %; TKIM 122, 89.7 %; TGENSCAN 133, 97.8 %; TFRAMA 129, 94.9 %). The assigned gene symbols, if present, were consistent with the TCUR annotation (Additional file 1: Table S10).

**Table 1:** Results of structural agreement of overlapping loci in the hetgla2 genome sequence

	Recovered^a^	Identical^b^	Matching^b^	Other^b^
TCUR (loci: 136)				
TFRAMA	129; 94.9 %	100; 77.5 %	15; 11.6 %	14; 10.9 %
TGNOMON	135; 99.3 %	114; 84.4 %	8; 5.9 %	13; 9.6 %
TKIM	122; 89.7 %	50; 41.0 %	16; 13.1 %	56; 45.9 %
TGENSCAN	133; 97.8 %	13; 9.8 %	6; 4.5 %	114; 85.7 %
TGNOMON (loci: 19,746)				
TFRAMA	14,387; 72.9 %	8463; 58.8 %	2127; 14.8 %	3797; 26.4 %
TKIM	14,933; 75.6 %	5382; 36.0 %	2647; 17.7 %	6904; 46.2 %
TGENSCAN	16,082; 81.4 %	1584; 9.8 %	1044; 6.5 %	13,454; 83.7 %

Each orthologous set of transcripts was compared to TCUR and TGNOMON, after filtering of alignments with perfectly aligned CDS (>99 % recovered in genome). CDSs are considered overlapping if they share nucleotides on the same strand. CDS overlap cases were classified to the following categories: identical (identical exons), matching (shared exons), or ‘other’ (unequal number of exons)

^a^Number of overlapping loci and their proportion of the loci in reference

^b^Number of identical, matching and other transcript models and their proportion of the loci in overlap

Next, we investigated the structural agreement between transcripts of the different transcript cataloging methods. Overlapping transcripts from different sources were classified based on the number and type of shared exons (Additional file 2: Figure S4): (i) identical transcripts have all exons exactly corresponding, (ii) matching transcripts share all exons, but not necessarily all exon boundaries, and (iii) others. Application of this classification scheme on TCUR loci showed that the proportion of identical and matching transcript models differed largely between genome-dependent methods (TGNOMON 122 of 135, 90.4 %; TKIM 66 of 122, 54.1 %; TGENSCAN: 19 of 133, 14.3 %). TFRAMA showed results close to TGNOMON (identical/matching 115; 89.1 %) and outperformed TKIM and TGENSCAN. Given that these primary results indicated superior quality of TGNOMON in respect to curated transcripts, we used it as a reference for a second, genome-wide quality assessment. According to this, TFRAMA resembles TGNOMON transcript models by showing the highest number of identical and matching loci (10,590; 73.6 %), in contrast to TKIM (8029; 53.8 %) and TGENSCAN (2628; 16.3 %). More specifically, TFRAMA also shows more transcript models identical to a TGNOMON counterpart (8463; 58.8 %) than TKIM (5382; 36.0 %). Together, this demonstrates a quality ranking of TGNOMON > TFRAMA > TKIM > TGENSCAN.

### Performance evaluation

The runtime of FRAMA mainly depends on the number of input reads, the resulting number of assembled transcript contigs and the size of the reference transcriptome. For the complete NMR dataset and 34,655 reference transcripts as input, FRAMA had a total runtime of 338 h on an 8-CPUs Linux workstation (Intel Xeon, 2.83 GHz, Model E5440) and a memory size of 32 GByte. The major computational load was due to de novo assembly and BLAST searches, each taking about 40 % of the total runtime. Using a smaller input subset of 40 million reads, the total run time of FRAMA decreased to 48 h, indicating that the total runtime linearly depends on the volume of the read data.

## Discussion

Though whole-genome sequencing and assembly is an essential prerequisite for genome-wide analyses, providing a plethora of information, it is still quite labor-intensive, time-consuming and costly. For example, three groups have independently worked on NMR genome assemblies and associated gene annotations, over the last four years [24, 25, 33]. In contrast, transcriptome sequencing and de novo transcriptome assembly is an affordable approach for first-pass sequence analysis of novel organisms, given automated concepts for extraction of transcripts from RNA-seq data. Towards this goal, we present FRAMA, an mRNA assembly and annotation pipeline for eukaryotes, which is designed to transform a primary transcriptome assembly into a comprehensive, but low-redundant, catalog of reconstructed mRNA sequences.

FRAMA is extensively guided by orthologous transcripts of a reference organism. Orthologs are used (i) for assignment of gene symbols to anonymous transcript contigs, (ii) for identification of representative transcripts from a complicated mixture of mRNA isoforms, and (iii) for refinement of representative transcripts, including scaffolding of fragmented transcript contigs, removal of likely intron contamination, and clipping of weakly supported 3’ ends. Given the high relevance of the reference organism, the primary question is what species should be used. Often, there will be a tradeoff between closely related species that have a relatively weak gene annotation on one hand, and more distantly related species with a more comprehensive annotation on the other hand. Applied to the NMR case, the closest-related model organism is the guinea pig (CDS similarity NMR/guinea pig 92.3 %, NMR/human 89.1 %, Additional file 1: Table S4), with an estimated divergence time of 41 Mya [33]. However, the guinea pig genome sequence is rather fragmentary, and the gene annotation is largely confined to the results of Ensembl and NCBI annotation pipelines, which are driven by gene prediction and homology inference. Human, with a divergence time of ca. 88 Mya [34], seems more challenging with regard to sequence similarity searches, but is outstanding in its extensive and experimentally based gene annotation. In fact, human as a homology reference for the NMR gave very satisfying results in this study (88.0 % recovered orthologs), which suggests that even organisms as distant as 100 Mya or more could serve as a reliable basis for ortholog inference. Consistent with this, a methodological survey showed that ortholog inference using a BBH scheme performs well in comparison to other assignment methods, irrespective of species distance [16].

The simplification of gene content via orthologous inference is to some extent artificial, since the ortholog-driven approach fails to identify species-specific paralogs - at best, they are misclassified as orthologs. However, the low-redundant transcript catalog is a comfortable starting point for identification of such species-specific paralogs. It is also clear that a transcript catalog based on RNA-seq will remain incomplete with respect to the total gene content of an organism. Since, even after sampling of multiple tissues and developmental stages, mRNAs with highly specific and restricted expression profiles will not be sufficiently covered. A good example that illustrates both, tissue-specific expression as well as species-specific paralogy, is the family of olfactory receptors (ORs). Humans have 388 functional OR genes, predominantly expressed in sensory neurons of the nasal mucosa, whereas rats have 1259 OR genes. Consistently, the subterranean NMR, which has an outstanding olfactory capacity, show signs of ongoing positive selection and expansion of the OR family, according to targeted genome resequencing [35]. An incompleteness of such tissue-specific transcripts may be acceptable if a limited set of tissues will be analyzed in subsequent studies, and the established gene catalog contains all the genes expressed in those addressed tissues. Furthermore, tissue-specific expression patterns are typically known from related organisms and rarely change during evolution [36]. Thus, even a limited gene catalog from selected tissues can be expected to be conclusive with respect to gene content.

A clear advantage of FRAMA is that it does not require genome data, allowing the study of non-model organisms with yet unknown genome sequence. When we analyzed the FRAMA results for the NMR, we obtained quality measures for the two available genome sequences, which further illustrate the independence of the transcriptome approach. Given a good correspondence on the sequence level (99.9 %), the NMR transcriptome provided exon sequences that filled genomic gap regions estimated to make up 1.0 % of the latest available genome sequence [24]. In addition, reconstructed mRNAs spanned 1695 gaps within genomic scaffolds, thereby driving genome assembly towards higher contiguity. Together, curated as well as FRAMA transcripts provided independent support for improvements made in NMR genome assemblies through the past years [24].

Modern genome annotation strategies incorporate RNA-seq data as experimental evidence for genes. As it had to be expected, FRAMA based on RNA-seq alone does not outperform qualified genome-based annotation strategies, like NCBI’s GNOMON pipeline, that use multiple sources of gene support in addition to transcriptome sequencing [11]. On the other hand, the FRAMA transcript catalog outperformed the ab initio gene prediction using GENSCAN and the annotation of the first NMR genome. Moreover, the FRAMA transcript catalog was close to the result of GNOMON with respect to structurally identical or matching transcript models (Table 1, Additional file 2: Figure S4). The latter can be considered as the currently best NMR genome annotation and is also well supported by an independent set of scientist-curated NMR transcripts (Table 1, dataset TCUR). Striking heterogeneities were found between different genome-based annotations, especially if one assumes that the same experimental evidence of RNA-seq data was used. The compared methods have similar sensitivity in recovery of gene loci, measured on the TCUR dataset, but the results differ largely on the gene structure level. However, such heterogeneities are in agreement with a recent benchmark study on genome-based RNA-seq transcript reconstruction [37].

## Conclusions

FRAMA realizes the *de novo* construction of a low-redundant transcript catalog for eukaryotes, including the extension and refinement of transcripts. Thereby, it delivers a compilation of transcripts which we regard suitable for comprehensive downstream analyses performed by biologists without bioinformatics expert support.

## Methods

For a full list of external software including versions and references see Additional file 1: Table S1.

### Tissue sampling

Samples from cerebellum, pituitary, thyroid, adrenal gland, kidney, skin, liver and ovary were collected from one female naked mole-rat from a previously established colony, kept at the Leibniz Institute for Zoo and Wildlife Research (IZW, Berlin) [38]. Hypothalamus and testis samples were obtained from a male animal of the same colony. Animal housing and tissue sampling was compliant with national and state legislation (breeding allowance #ZH 156; ethics approval G 0221/12 “Exploring long health span”, Landesamt für Gesundheit und Soziales, Berlin).

### RNA-seq

Prior to RNA isolation, tissue was disrupted in the homogenization buffer of the RNA extraction protocol using a Tissue Lyser instrument (Qiagen). RNA was isolated using the RNeasy Mini kit (Qiagen), performing specialized protocols for brain and muscle tissues as recommended by the manufacturer. The RNA was treated with DNase I on the affinity column before elution. Strand specific RNA-seq libraries, including poly-A(+) mRNA selection and RNA fragmentation, were prepared using the TruSeq Stranded RNA LT Kit (Illumina) according to the supplier’s instructions, with 2 μg total RNA as input. The resulting libraries had insert sizes of ca. 100–400 bp as indicated by DNA 7500 Chips run on an Agilent Bioanalyzer 2100 instrument (Agilent). All ten libraries were combined into a single pool. Sequencing of 200-nt paired-end reads was performed using an Illumina HiSeq 2500 apparatus in Rapid mode with TruSeq Rapid SBS chemistry on two lanes (Illumina). Read data for each library were extracted in FastQ format using the CASAVA software v1.8.4 (Illumina) using default settings.

### Read preprocessing

Quality of RNA-seq reads was inspected using FastQC. Raw data was screened for potential cross-contamination with foreign species, including human, pig, mouse and guinea pig. Overlapping paired-end reads were joined into single longer reads (93.8 %), and adapter sequences of these and remaining reads were clipped using SeqPrep (parameters: −A < adaptrev > −B < adaptfwd>). Non-overlapping reads were quality-trimmed at the 3’ end using sickle (parameters: −x -q 23 -l 35), and reads shorter than 35 bp were discarded. Reverse-complemented antisense reads and sense reads were pooled with joined long reads to generate a set of stranded single reads (simply “reads” in the following).

### Reference sequence sets

Human transcripts, used as the reference for transcriptome reconstruction, were part of the human genome annotation release 105 obtained from the National Center for Biotechnology Information (NCBI). Selection for known protein-coding Reference Sequences (RefSeqs; NM-style accessions) resulted in 34,655 transcripts. Public human RNA-seq data (Illumina Body Map 2.0, Illumina Corp., unpublished) were used to assess mRNA expression. Mouse protein-coding RefSeqs were part of the mouse genome annotation release 104 obtained from NCBI (77,610 transcripts). NMR genome assemblies were previously reported by Kim et al. [24] (Bioproject: PRJNA68323; hetgla1) and Keane et al. [25] (Bioproject: PRJNA72441; hetgla2). The most recent hetgla2 genome sequence was used as the reference unless stated otherwise. Four sets of NMR transcripts from different sources were used for comparison: 76,826 Reference Sequence mRNAs modeled by NCBI’s eukaryotic genome annotation pipeline, GNOMON (NCBI Heterocephalus glaber Annotation Release 100; abbreviated as TGNOMON); 21,771 CDSs published by Kim et al. [24] (Bioproject: PRJNA68323; abbreviated as TKIM); 55,730 GENSCAN predictions obtained from UCSC (abbreviated as TGENSCAN); and 142 curated mRNA sequences obtained from GenBank (Additional file 1: Table S2; abbreviated as TCUR).

### Read alignment

Spliced alignment of the RNA-seq reads against the genome sequence was performed with STAR allowing 2 % mismatches within the aligned region and a maximum of 5 multiple hits per read (parameters: −outSAMstrandField intronMotif --outFilterMultimapNmax 5 --outFilterMismatchNoverLmax 0.02). RNA-seq read counts per gene were obtained via mapping with BOWTIE; per gene, the longest transcript was used as mapping template, and unique hits for each read were required. A comparison of human samples, based on expression values scaled to fragments per kb transcript per million fragments (FPKM) [39], was done using the Mann–Whitney *U*-test (two-sided), and p-values were obtained via a Monte Carlo-based approximation implemented in the R package COIN.

### Multiple sequence alignment

For orthologous assignment of CDS we created a resource of multi-species mRNA alignments. Starting with the reference mRNAs of human, dog, mouse, and rat (NCBI RefSeq, release 61), orthologous clusters were identified using the HomoloGene database (release 67) [40]. Multiple protein sequence alignments for each cluster were computed using CLUSTALW (parameter: gapext = −2). For each human isoform, a sub-alignment was extracted from the orthologous cluster, such that the one most similar isoform from each of the other species was contained.

### Analysis of transcript-to-genome alignments

Quality of transcript sequence sets was assessed from transcript-to-genome alignments. The following approach was applied to all transcript sets to ensure equal conditions. Transcript sequences were mapped with BLAT (parameter: −extendThroughN) and filtered for one global best hit using the BLAT utility pslCDnaFilter (parameters: −globalNearBest = 0.0 -minAlnSize = 100 -minId = 0.9). Spliced alignment was determined with SPLIGN (parameters: −gap_extension_score −520 -type est -direction sense -min_exon_idty 0.85 -min_compartment_idty 0.6) within the best BLAT hit region including 1 kb up- and downstream. Poorly aligned regions were determined with an in-house implemented hidden Markov model, which identifies regions of significantly high mismatch density due to lack of appropriately aligning genome regions.

An all-against-all comparison between gene annotations was used to determine shared genes and transcripts. Briefly, within a gene annotation, genes are defined either by single-transcript loci or by multiple transcripts overlapping on the same strand. One-to-one relationships between transcripts from different annotations were calculated with EVALUATOR.pl, which utilizes a stable marriage algorithm to pair transcripts for each gene locus. The number of overlapping, missing or wrong exons was determined with in-house software. The structural agreement was investigated for the CDS of transcripts with perfectly aligned CDS (>99 % aligned).

### Data access

RNA-seq data and assembled transcripts with full-length CDS were deposited at NCBI databases (linked to Bioproject PRJNA283581). FRAMA is available for download at https://github.com/gengit/FRAMA.

## Availability and requirements

Project name: FRAMA (from RNA-seq to annotated mRNA assembly)

Project home page: https://github.com/gengit/FRAMA

Operating System: UNIX/Linux

Programming language: Perl, R

Other requirements: Additional file 1: Table S1 and https://github.com/gengit/FRAMA.

License: FLI-Licence

### Availability of supporting data

Additional file 1: Supplementary Tables.

Additional file 2: Supplementary Figures.

## Additional files

Additional file 1: Table S1. List of external software. **Table S2**: NMR transcript data set TCUR, and orthologous transcripts from human, mouse and guinea pig. Multi-species mRNA alignments were constructed independently from those described in the main text, using the sequence database entries as listed. **Table S3**: Naked mole-rat samples for strand-specific RNA-seq, and produced RNA-seq data. **Table S4**: Pairwise transcript sequence identities between NMR and related mammals. The analysis is based on 142 multiple sequence alignments of the CDSs of NMR, guinea pig, human and mouse (as listed in Additional file 1: Table S2). Identity values were computed based on gap-masked alignments. **Table S5**: Statistics of the transcriptome data produced by Trinity (column “transcript assembly”) and subsequently processed using FRAMA (column “transcript catalog”). **Table S6**: CEGMA results on transcriptome datasets. As defined by CEGMA, ‘complete proteins’ are recovered with >70 % in comparison to CEGMA’s core proteins. ‘Partial proteins’ additionally include proteins, which exceed a certain alignment score threshold. CEGMAs software components were used as suggested: geneid (v1.4), genewise (wise2.2.3-rc7), hmmer (HMMER 3.0), NCBI BLAST+ (2.2.25). **Table S7**: Source of transcript sequence sets and underlying input data. **Table S8**: Transcript-genome alignment statistics of curated dataset (TCUR) in hetgla1. The alignments comprise 1473 well-aligned blocks and 81 unaligned or mismatching blocks. Transcripts show 99.9 % average identity within well-aligned blocks. **Table S9**: Transcript-genome alignment of curated dataset (TCUR) in hetgla2. The alignments comprise 1525 well-aligned blocks and 16 unaligned or mismatching blocks. Transcripts show 99.9 % average identity within well-aligned blocks. **Table S10**: Correspondence of gene symbols between transcript sets. The evaluation considered gene loci overlapping in the hetgla2 genome sequence, where all transcript-genome alignments of a gene were considered to define the gene locus. Only genes with ascertained function (non-LOC gene symbol) were compared. **Table S11**: Accession numbers of sequences that are shown in the genome-based transcript map (hetgla2, scaffold JH602043; Fig. 4). Accession numbers for each sequence are listed in the same order as shown in Fig. 4 (from top to bottom). (XLSX 77 kb) Additional file 2: Figure S1. Multiple sequence alignments of CALM1, CALM2 and CALM3 in human and NMR. **(A)** protein coding sequence **(B)** protein sequence. All protein coding sequences encode for the same protein sequence. The nucleotide identity between human and NMR orthologs is higher (97 % CALM1, 98 % CALM2, 95 % CALM3) than the intra-species paralog identity (e.g., human CALM1/CALM2 highest identity with 85 %). **Figure S2**. Recovery of transcripts is predicted by the expression level in the reference organism - **(A)** human liver, **(B)** human kidney. Public human Illumina RNA-seq data were obtained from the Short Read Archive at the EBI (accessions ERR030895 and ERR030893, respectively). Box plots show the human expression levels in log-scale FPKM; zero FPKM values were initially transformed to 0.80 times the lowest finite value. Human genes are displayed in three groups: all genes (“all”), genes recovered as orthologous NMR transcripts (“recovered”), and genes missing in the NMR transcript catalog (“missing”). Boxes enclose the data ranges of the central two-third quantiles, and central bars indicate the data medians. Note that the group-wise medians are significantly influenced by the fraction of zero-expression genes; these are 12 % in the liver-recovered group, 56 % in the liver-missing group, 7 % in the kidney-recovered group, and 49 % in the kidney-missing group. **Figure S3**. Results of structural agreement between transcript sets. The evaluation considered gene loci overlapping in the hetgla2 genome. Each transcript set was compared to TCUR. **Figure S4**. Classification of exons into four categories (exact, overlapping, missing and wrong) based on the reference transcript model. Exact exons share the same boundaries. Overlapping exons share base pairs, but not necessarily any boundary. Exons only present in the predicted transcript model are classified as wrong. Exons only present in the reference transcript model are classified as missing. (DOCX 841 kb)

## Abbreviations

BBH: best bidirectional blast hit
CDS: protein-coding sequence
MSA: multiple sequence alignment
NMR: naked mole-rat
RNA-seq: second-generation sequencing of RNA
SBH: single best blast hit
UTR: untranslated regions
