# Supplementary material for: FRAMA: from RNA-seq data to annotated mRNA assemblies
Source: BMC Genomics. 2016 Jan 14;17:54. doi: 10.1186/s12864-015-2349-8 (PMC4712544; doi:10.1186/s12864-015-2349-8)

**Additional file 2**

**Figure S1**: Multiple sequence alignments of CALM1, CALM2 and CALM3 in human and NMR. **(A)** protein coding sequence **(B)** protein sequence. All protein coding sequences encode for the same protein sequence. The nucleotide identity between human and NMR orthologs is higher (97% CALM1, 98% CALM2, 95% CALM3) than the intra-species paralog identity (e.g. human CALM1/CALM2 highest identity with 85%).

**
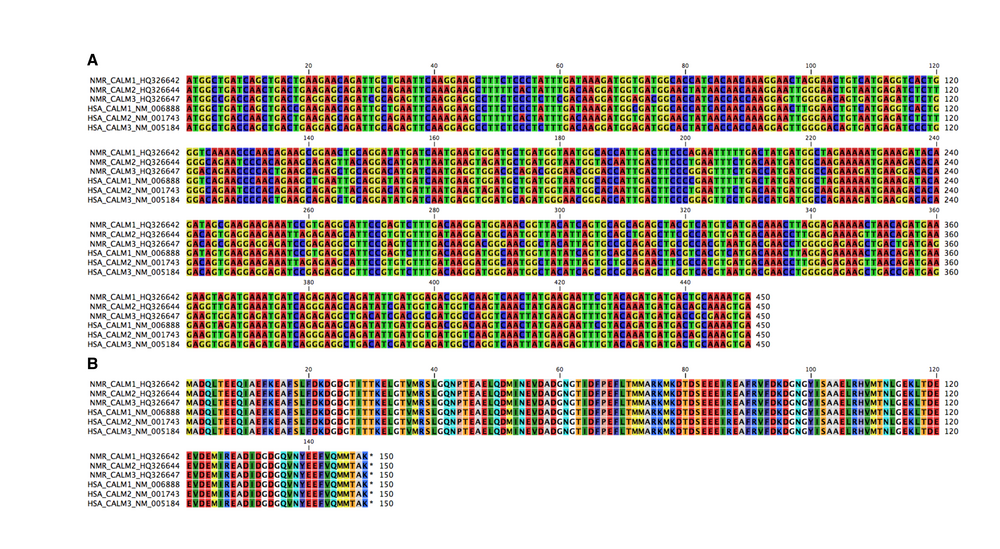
**

**Figure S2.** Recovery of transcripts is predicted by the expression level in the reference organism - **(A)** human liver, **(B)** human kidney. Public human Illumina RNA-seq data were obtained from the Short Read Archive at the EBI (accessions ERR030895 and ERR030893, respectively). Box plots show the human expression levels in log-scale FPKM; zero FPKM values were initially transformed to 0.80 times the lowest finite value. Human genes are displayed in three groups: all genes (“all”), genes recovered as orthologous NMR transcripts (“recovered”), and genes missing in the NMR transcript catalog (“missing”). Boxes enclose the data ranges of the central two-third quantiles, and central bars indicate the data medians. Note that the group-wise medians are significantly influenced by the fraction of zero-expression genes; these are 12% in the liver-recovered group, 56% in the liver-missing group, 7% in the kidney-recovered group, and 49% in the kidney-missing group.


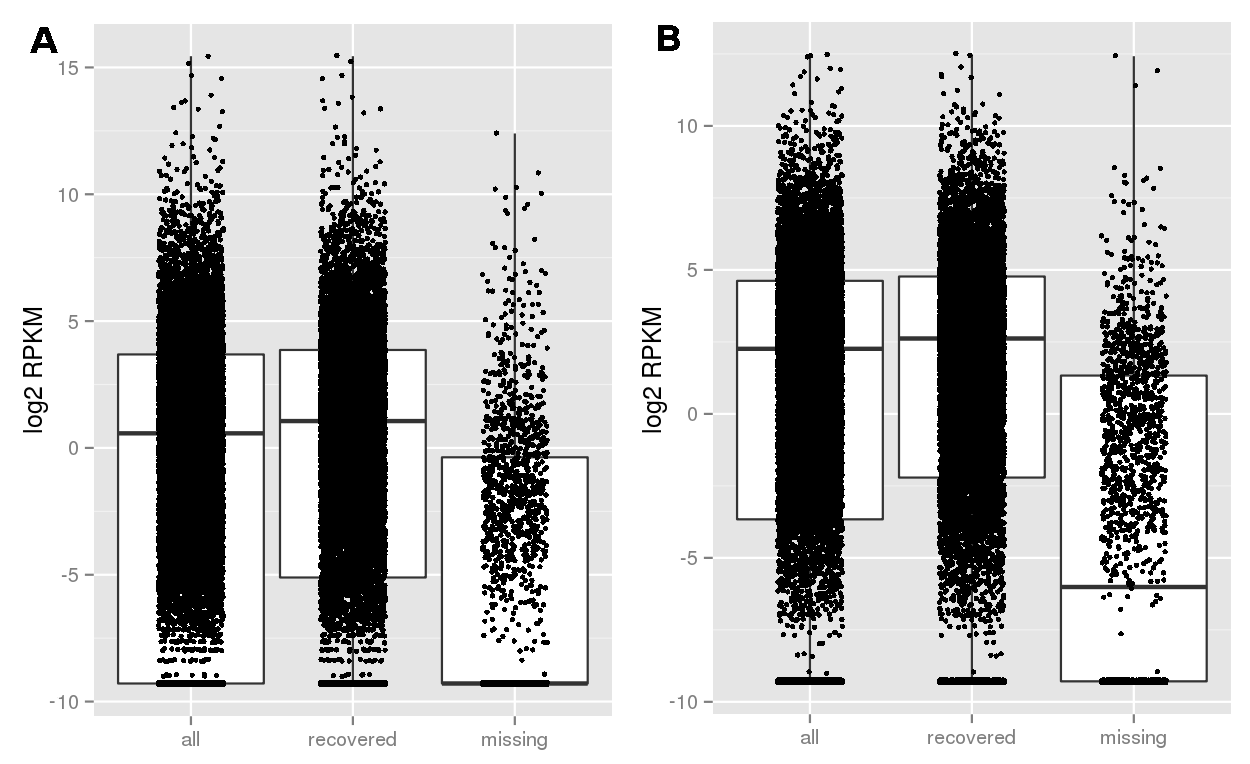


**Figure S3**: Results of structural agreement between transcript sets. The evaluation considered gene loci overlapping in the hetgla2 genome. Each transcript set was compared to TCUR.


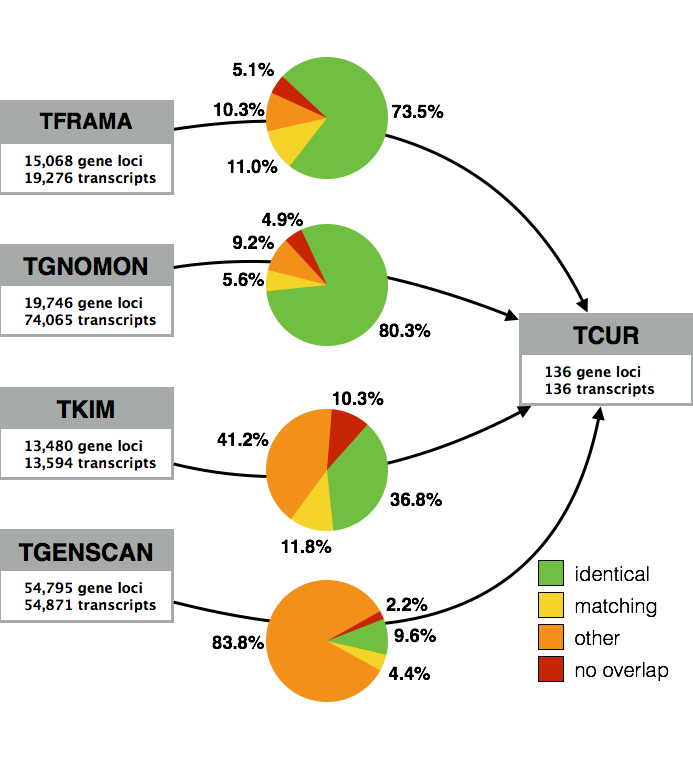


**Figure S4**: Classification of exons into four categories (exact, overlapping, missing and wrong) based on the reference transcript model. Exact exons share the same boundaries. Overlapping exons share base pairs, but not necessarily any boundary. Exons only present in the predicted transcript model are classified as wrong. Exons only present in the reference transcript model are classified as missing.


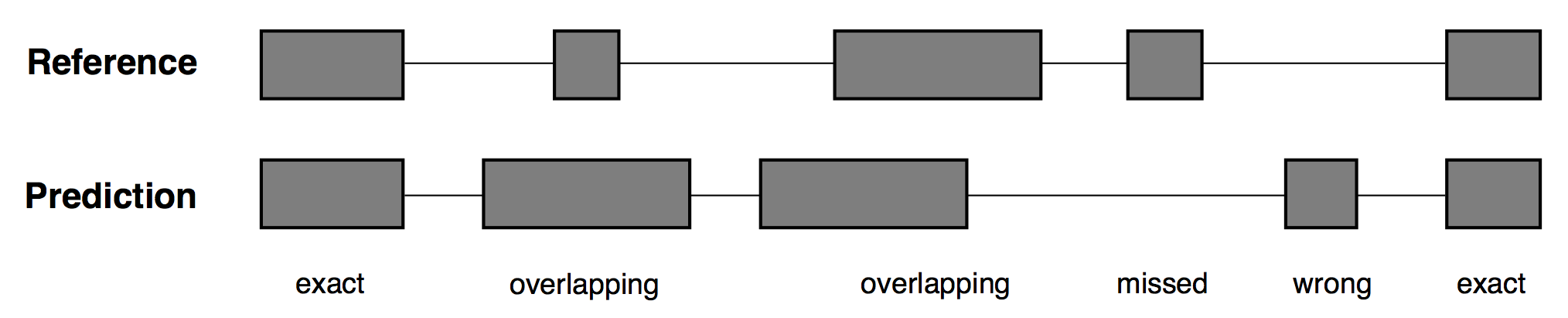

Supplement: Additional file 2: Figure S1. — Multiple sequence alignments of CALM1, CALM2 and CALM3 in human and NMR. (A) protein coding sequence (B) protein sequence. All protein coding sequences encode for the same protein sequence. The nucleotide identity between human and NMR orthologs is higher (97 % CALM1, 98 % CALM2, 95 % CALM3) than the intra-species paralog identity (e.g., human CALM1/CALM2 highest identity with 85 %). Figure S2. Recovery of transcripts is predicted by the expression level in the reference organism - (A) human liver, (B) human kidney. Public human Illumina RNA-seq data were obtained from the Short Read Archive at the EBI (accessions ERR030895 and ERR030893, respectively). Box plots show the human expression levels in log-scale FPKM; zero FPKM values were initially transformed to 0.80 times the lowest finite value. Human genes are displayed in three groups: all genes (“all”), genes recovered as orthologous NMR transcripts (“recovered”), and genes missing in the NMR transcript catalog (“missing”). Boxes enclose the data ranges of the central two-third quantiles, and central bars indicate the data medians. Note that the group-wise medians are significantly influenced by the fraction of zero-expression genes; these are 12 % in the liver-recovered group, 56 % in the liver-missing group, 7 % in the kidney-recovered group, and 49 % in the kidney-missing group. Figure S3. Results of structural agreement between transcript sets. The evaluation considered gene loci overlapping in the hetgla2 genome. Each transcript set was compared to TCUR. Figure S4. Classification of exons into four categories (exact, overlapping, missing and wrong) based on the reference transcript model. Exact exons share the same boundaries. Overlapping exons share base pairs, but not necessarily any boundary. Exons only present in the predicted transcript model are classified as wrong. Exons only present in the reference transcript model are classified as missing. (DOCX 841 kb) [file 12864_2015_2349_MOESM2_ESM.docx]
